# Dynamic Alterations in DNA Methylation of CD4^+^ T Cells and Macrophages in a Murine Model of Tuberculous Pleural Infection Induced by BCG Vaccination

**DOI:** 10.1002/mco2.70166

**Published:** 2025-04-01

**Authors:** Ming‐Ming Shao, Qing‐Yu Chen, Xin Zhang, Shu‐Feng Dong, Rui‐Qi Wei, Huan‐Zhong Shi, Feng‐Shuang Yi

**Affiliations:** ^1^ Department of Respiratory and Critical Care Medicine Beijing Institute of Respiratory Medicine and Beijing Chao‐Yang Hospital Capital Medical University Beijing China; ^2^ Medical Research Center Beijing Institute of Respiratory Medicine and Beijing Chao‐Yang Hospital Capital Medical University Beijing China

**Keywords:** BCG vaccination, C1q, CD4^+^ T cells, DNA methylation, Macrophages, tuberculous pleural infection

## Abstract

Tuberculous pleural effusion (TPE) is a prevalent form of extrapulmonary tuberculosis, and immune abnormalities play a crucial role in promoting its development. However, the dynamic changes and regulatory characteristics of immune cells during TPE progression remain incompletely understood. This study analyzed DNA methylation and transcriptome data from macrophages and CD4^+^ T cells from pleural lavage fluid of BCG‐induced tuberculous pleurisy mouse models at specific time points (Days 0, 1, 7, and 14). The results revealed substantial alterations in DNA methylation patterns associated with inflammatory factors and interferon genes. Notably, macrophages exhibited the most pronounced differences in DNA methylation profiles on Day 1, while CD4^+^ T cells demonstrated gradual changes over time. The investigation further indicated that DNA methylation primarily regulated the differentiation of Th1, Th17, and Th22 cells but not Th9 cells. Additionally, single‐cell RNA sequencing analysis revealed an increasing expression of C1q during infection, which was regulated by DNA methylation. Importantly, C1q^+^ and C1q^−^ macrophages demonstrated distinct roles in modulating immune responses during infection. This research provides valuable insights into the DNA methylation profile of immune cells during *Mycobacterium bovis* infection–induced pleurisy in a mouse model, enhancing our understanding of the upstream regulatory mechanisms underlying immune response development in TPE.

## Graphical abstract

We obtained DNA methylation and transcriptome data of macrophages and CD4^+^ T cells isolated from pleural lavage fluid of BCG‐induced tuberculous pleurisy mouse models at Days 0, 1, 7, and 14. DNA methylation primarily controlled the differentiation of Th1 and Th17 cells, polarization, and increasing C1q expression of macrophages. C1q^+^ macrophages have less interaction with effector T cells and exhibit immunosuppressive characteristics.

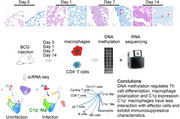

## 1 Introduction

Tuberculosis (TB) remains a global public health concern that poses a significant threat to human health. According to the World Health Organization's report, in 2021, over 10 million TB cases were recorded worldwide, with 1.6 million deaths (WHO report). Tuberculous pleural effusion (TPE), characterized by the accumulation of inflammatory cells in the pleural cavity, is one of the most prevalent forms of extrapulmonary TB [1]. TPE patients frequently experience symptoms such as chest pain, breathlessness, and cough, which substantially impact their quality of life [2]. The establishment of mouse models of pleurisy through intrapleural injection of BCG vaccine has been widely adopted [3, 4]. Investigating the dynamic immune regulation in BCG‐induced mouse pleurisy models presents a promising approach to identifying novel targets for TB protection.

During the progression of TB, innate and adaptive immune cells collaborate to control *Mycobacterium tuberculosis* infection [5, 6]. Following exposure to *M. tuberculosis* via aerosol transmission, the host initiates an innate immune response to combat the infection. Macrophages, pivotal components of the innate immune system, engulf inhaled *M. tuberculosis* and play a crucial role in shaping the subsequent immune response. However, their function during *M. tuberculosis* infection is complex, sometimes contributing to host resistance and at other times facilitating infection establishment [7, 8]. Adaptive immune cells are primed and activated after the innate immune cell response, typically taking 2 weeks or longer to establish. CD4^+^ helper T cells are critical for containing *M. tuberculosis* growth [5]. Adaptive immune cells, such as Th1, Th17, and B cells, secrete cytokines like IFN‐γ, IL‐17, IL‐22, and antibodies to participate in *M. tuberculosis* clearance [9]. Similar to macrophages, not all T cell responses to *M. tuberculosis* contribute to protecting humans from the disease; the balance between infection control and inflammation restraint is critical to the outcome of the infection [6, 10, 11, 12]. BCG remains a widely and frequently used vaccine against TB, with CD4^+^ T cell responses, including the production of IFN‐γ, IL‐17, and TNF, serving as candidates to evaluate protection following BCG vaccination [13]. Despite effective cooperation between innate and adaptive immune systems, which usually results in latent TB infection in most individuals, a subset of vaccinated individuals (approximately 5%–10%) still develop active TB. The underlying mechanisms for this variable outcome remain poorly understood [9]. Although BCG vaccination is an effective method for preventing TB among children and adolescents, its protection among adults remains a subject of debate [14, 15].

DNA methylation, a chemical modification to DNA, regulates gene expression and genome stability, and its disruption is associated with various diseases, including TB [16, 17]. DNA methylation analysis serves as a powerful technique to investigate the TB spectrum. Research has shown that *M. tuberculosis* infection modulates the transcriptional profiles of macrophages and CD4^+^ T cells, enabling evasion of host defense mechanisms by altering the methylation of genes crucial for immune response [18]. Significant alterations in immune‐regulating genes and pathways due to DNA methylation have been observed in CD4^+^ T cells following BCG vaccination [19]. While numerous studies have examined host immune responses to *M. tuberculosis* from the perspective of DNA methylation, limited research has focused on dissecting the methylation status at different stages during TB progression, particularly in TPE. Investigating methylation patterns at various time points during TB infection could yield valuable insights into the disease's pathogenesis and potentially identify novel targets for therapeutic intervention. Further research in this area may contribute to the development of more effective TB vaccines or therapeutics targeting epigenetic modifications such as DNA methylation.

Employing advanced techniques of high‐throughput DNA methylation profiling and next‐generation sequencing, we conducted a comprehensive analysis integrating mRNA expression, methylation patterns, and their associated transcriptional pathways. This analysis focused on macrophages and CD4^+^ T cells isolated from mouse pleural lavage fluid at critical time points: Days 1, 7, and 14 following BCG injection. By comparing these findings to baseline data collected on Day 0 (prior to BCG pleural injection), we constructed a novel and detailed portrait of innate and adaptive immune regulation–related gene expression as the infection progressed over time.

## 2 Results

### 2.1 DNA Methylation Aberrations in BCG‐Induced Tuberculous Pleurisy Mouse Models

**FIGURE 1:**
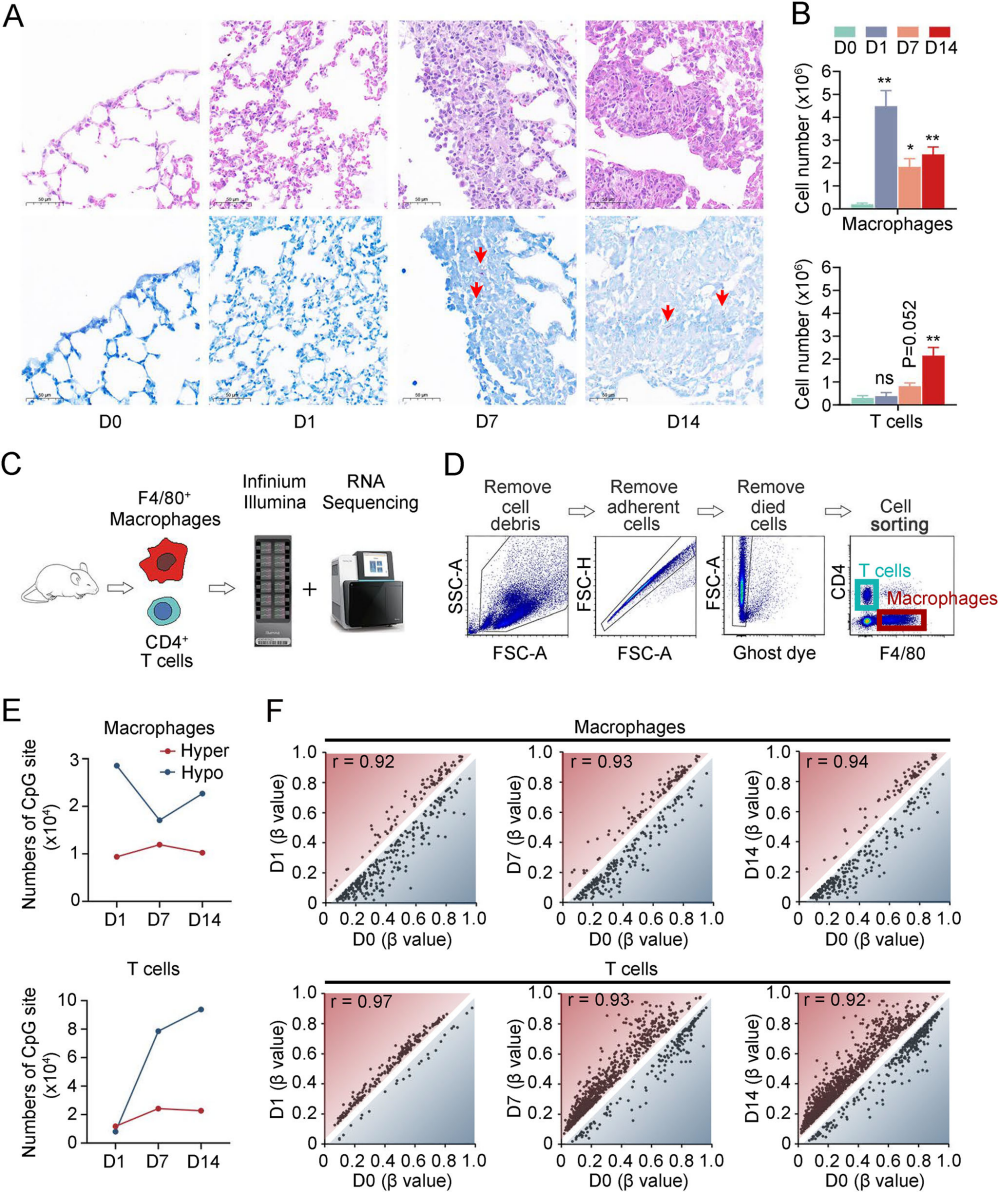
DNA methylation analysis of BCG‐induced tuberculous pleurisy mouse models. (A) Hematoxylin and eosin (H&E) stains (upper panel) and acid‐fast bacteria stain (bottom panel of lung tissue of BCG‐induced tuberculous pleurisy mouse models at different time points (*n* = 5). (B) Cell number of macrophages (upper panel) and CD4^+^ T cells (bottom panel) in pleural lavage fluid of BCG‐induced tuberculous pleurisy mouse models at different time points (*n* = 5). Data are presented as means ± SEM. *p < 0.05, **p < 0.01; the comparisons were determined by Student's *t*‐test. (C) Sample processing pipeline in the study. (D) Gating strategy of macrophage and CD4^+^ T cell sorting by flow cytometry. (E) The numbers of hyper‐ or hypo‐CpG sites under the three groupwise comparisons in macrophages (upper panel) and CD4^+^ T cells (bottom panel). (F) Correlation analysis of methylation levels at corresponding time.

Guided by the leukocyte accumulation profile observed during BCG‐induced tuberculous pleurisy in mice, we selected specific time points for detailed analysis: Day 0 (prior to BCG injection) and Days 1, 7, and 14 following BCG injection. Histological examination revealed inflammatory cell infiltration in the lungs as early as Day 1 post‐BCG injection, with progressive accumulation observed at Days 7 and 14 (Figure 1A). AFB stain experiments demonstrated that bacilli were scarcely detectable in mouse lung tissue at Day 1 but easily observed at Days 7 and 14 (Figure 1A). Additionally, we collected mononuclear cells from pleural lavage fluid (Table S1). During the process of TB, innate immune macrophages and adaptive immune CD4^+^ T lymphocytes play crucial roles in controlling infection. At Day 1, a significant increase in macrophage numbers was observed. Subsequently, the macrophage count decreased and maintained a relatively stable intermediate level at Days 7 and 14 (Figure 1B). In contrast, T lymphocytes showed no significant change in cell number at Day 1 compared to Day 0. However, following BCG injection, the number of T lymphocytes gradually increased, with the cell count more than doubling by Day 14 compared to Day 7 (Figure 1B).

DNA methylation plays a crucial role in regulating gene expression and functions across various diseases, including cancer, immunological and neurological disorders, and infections. To investigate methylation status and RNA expression levels in these processes, we isolated macrophages and CD4^+^ T lymphocytes from infected mice and utilized mouse methylation chips and next‐generation RNA sequencing (Figures 1C and S1A). CD4^+^ T lymphocytes and F4/80^+^ macrophages were isolated through flow cytometry (Figure 1D). Principal component analysis revealed distinct DNA methylation profiles for macrophages and CD4^+^ T cells (Figure S1B). In macrophages, the number of hypomethylated CpG sites decreased by half on Day 7 and increased slightly on Day 14 compared to uninfected samples, while hypermethylated CpG site numbers remained relatively stable. Conversely, both hyper‐ and hypomethylated CpG site numbers gradually increased in CD4^+^ T cells postinfection. Notably, the number of hypomethylated CpG sites was nearly 10 times higher on Day 14 than on Day 1 (Figure 1E). Concurrently, as the infection persisted, the correlation coefficient between macrophages' methylation profile and the uninfected samples gradually increased (D1–D0, *r* = 0.92; D7–D0, *r* = 0.93; D14–D0, *r* = 0.94; All *p* < 0.001), while the correlation coefficient between CD4^+^ T cell methylation profile and uninfected samples decreased (D1–D0, *r* = 0.97; D7–D0, *r* = 0.93; D14–D0, *r* = 0.92; All *p* < 0.001) (Figure 1F). These subtle changes in genome‐wide DNA methylation profile correlation coefficients represent significant methylation alterations across thousands of CpG sites. During the continuous infection of BCG‐induced tuberculous pleurisy mouse models, the difference in DNA methylation profile between macrophages and uninfected tissues was most pronounced on Day 1, while the difference between CD4^+^ T cells and uninfected tissues became increasingly evident over time. This observed phenomenon aligns with the established understanding that innate immune cells respond more rapidly to pathogen invasion compared to adaptive immune cells, with the latter requiring up to 2 weeks or longer for the establishment of an adaptive immune response.

### 2.2 DNA Methylation Was a Regulatory Factor of Th Cell Differentiation

**FIGURE 2:**
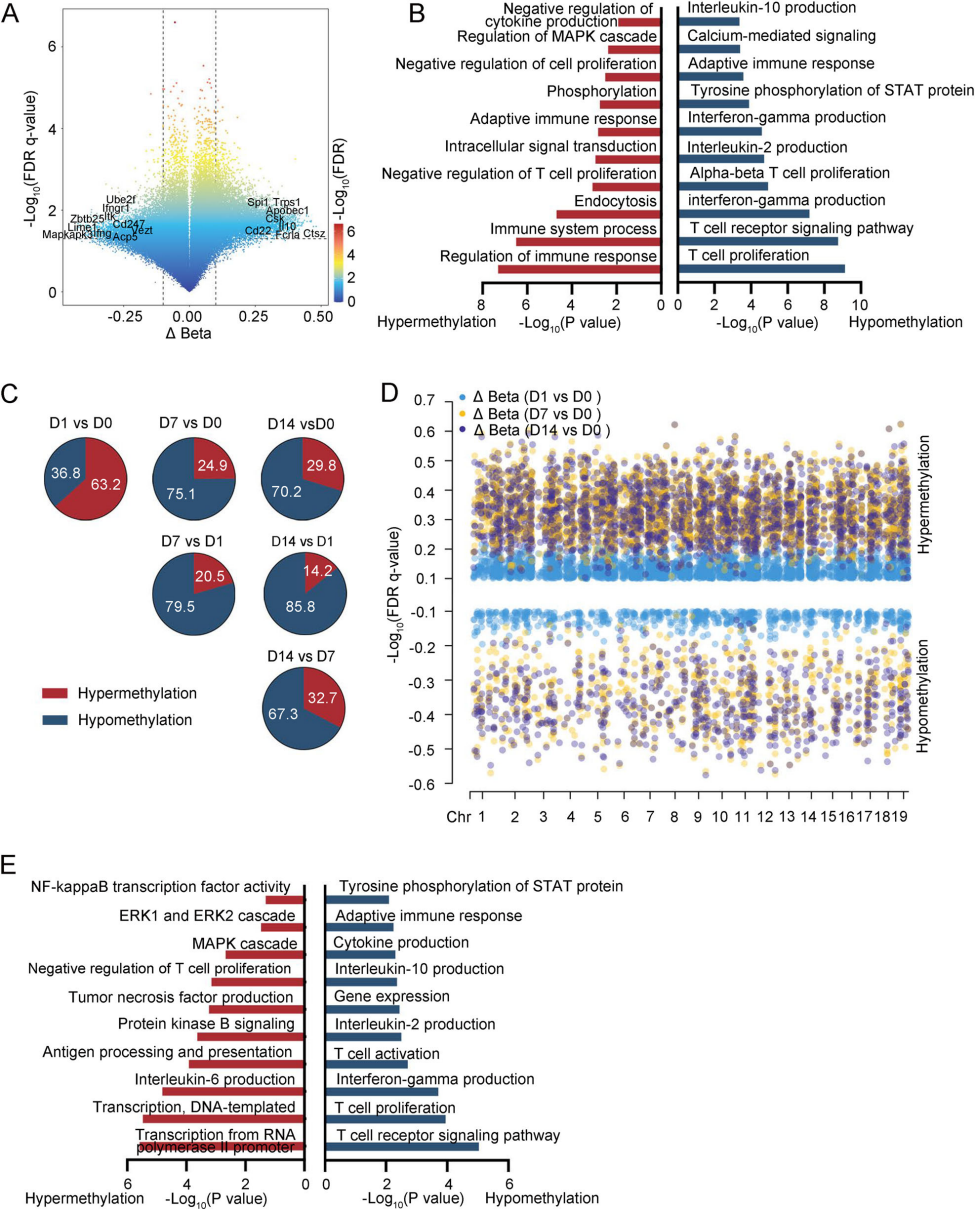
The differential methylated cytosines (DMCs) in CD4^+^ T cells between BCG‐induced tuberculous pleurisy mouse models and control. (A) Volcano plot showed the DMCs of CD4^+^ T cells in pleural lavage fluid of mice with BCG‐induced tuberculous pleurisy and those in untreated mice. The host genes for the partial differential DMCs are depicted. (B) Pathway enrichment analysis of DMCs of CD4^+^ T cells in pleural lavage fluid of mice with BCG‐induced tuberculous pleurisy and those in untreated mice. (C) Pie charts showed the status of the DMCs identified in groupwise comparisons. Numbers show the frequency of hyper‐ or hypomethylation. (D) The combined Manhattan plot showed the shared DMCs on Days 1, 7, and 14 of infection in BCG‐induced tuberculous pleurisy mouse models compared to Day 0, respectively. Hypermethylated and hypomethylated DMCs were divided into upper and lower sides of the Manhattan plot, respectively. (E) Pathway enrichment analysis of the shared DMCs on Days 1, 7, and 14 of infection in BCG‐induced tuberculous pleurisy mouse models compared to Day 0, respectively.

This study conducted a comprehensive analysis of DNA methylation levels and their correlation with gene functions in macrophages and CD4^+^ T cells infected with BCG. In CD4^+^ T cells, a total of 15,133 methylation sites differed from the uninfected samples. These methylation sites were predominantly associated with demethylation events, which were enriched in various processes including interleukin‐10 production, adaptive immune response, tyrosine phosphorylation of STAT protein, interferon‐gamma production, interleukin‐2 production, alpha–beta T cell proliferation, T cell receptor signaling pathway, and T cell proliferation signaling pathway. These findings are consistent with previous research in human TPE (Figure 2A,B). Except for the first day, the changes in DNA methylation sites on subsequent days were primarily characterized by demethylation compared to the preceding days (Figures 2C and S2A). To elucidate the immunological commonalities in macrophages during persistent BCG infection, we performed differential analysis both pairwise and longitudinally in the methylation profile. These differential methylated cytosines (DMCs) shared by CD4^+^ T cells exhibited a predominant hypermethylation pattern, mainly affecting pathways involved in the negative regulation of CD4^+^ T cell proliferation and tumor necrosis factor production (Figure 2D,E).

**FIGURE 3:**
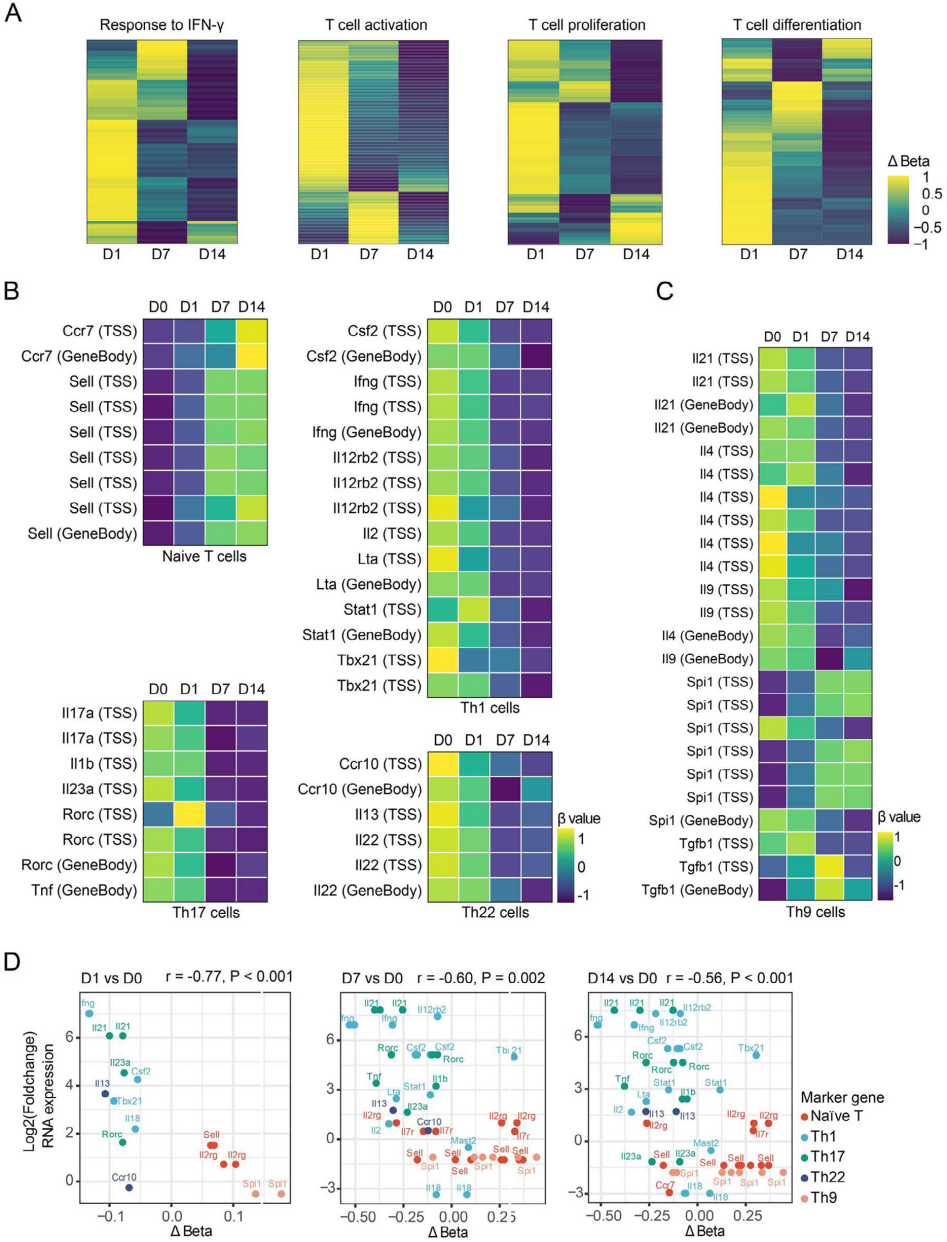
DNA methylation–regulated CD4^+^ T cell differentiation. (A) Heatmaps showed the differential methylation level of the genes in the corresponding pathways. (B) Heatmaps showed the methylation level of the marker genes of naïve CD4^+^ T cells, Th1 cells, Th17 cells, and Th22 cells. Each row represents a methylation site. (C) Heatmaps showed the methylation level of the marker genes of Th9 cells. Each row represents a methylation site. (D) Correlation analyses visualized the relation between differential methylation level (Δbeta) and associated gene expression (log_2_ fold‐change, *y*‐axis) in marker genes of CD4^+^ T cell subtypes.

We subsequently investigated the relationship between the methylation status of CD4^+^ T cells and their functional properties, particularly regarding T cell differentiation. Utilizing the GO database, we identified a panel of genes involved in major T cell anti‐*M. tuberculosis* immune response signaling pathways, including IFN‐γ response, T cell activation, proliferation, and differentiation. Heatmap analysis revealed that demethylation levels in these genes tended to increase over time (Figure 3A). To further elucidate the methylation patterns in distinct T cell subsets, we examined methylation levels associated with characteristic cytokines and transcription factors in naïve CD4^+^ T cells, as well as Th1, Th17, Th9, and Th22 cells, which play a pivotal role in human TPE infection immunity. As BCG infection persisted, we observed increased methylation levels in markers of naïve CD4^+^ T cells, such as Ccr7 and Sell. Concurrently, there was a decrease in methylation levels for markers of Th1 cells (Csf2, Ifng, Lta, Stat1, and Tbx21), Th17 cells (Il17a, Rorc, and Tnf), and Th22 cells (Ccr10 and Il22) (Figure 3B). However, the methylation patterns of Th9 cell markers like Il9 and Spi1 exhibited inconsistent changes (Figure 3C). Subsequently, we analyzed the correlation between the methylation status and expression levels of genes in CD4^+^ T cell subsets. As shown in Figure 3D, we observed a significant negative correlation between the methylation levels of these signature genes and their corresponding gene expression. Notably, with the prolongation of BCG infection, the demethylation sites of genes regulating Th1 and Th17 cell differentiation increased most prominently. Interestingly, the expression level of the Spi1 gene did not correlate with its demethylation status (*p* = 0.82). These findings suggest that in BCG‐induced tuberculous pleurisy mouse models, the degree of demethylation in genes related to CD4^+^ T cell activation, proliferation, and differentiation generally increases with the progression of infection. DNA methylation levels significantly influenced the differentiation of Th1, Th17, and Th22 cells, although methylation might not serve as the primary regulatory mechanism governing Th9 cell differentiation.

### 2.3 The DMCs in Macrophages Between BCG‐Induced Tuberculous Pleurisy Mouse Models and Control

**FIGURE 4:**
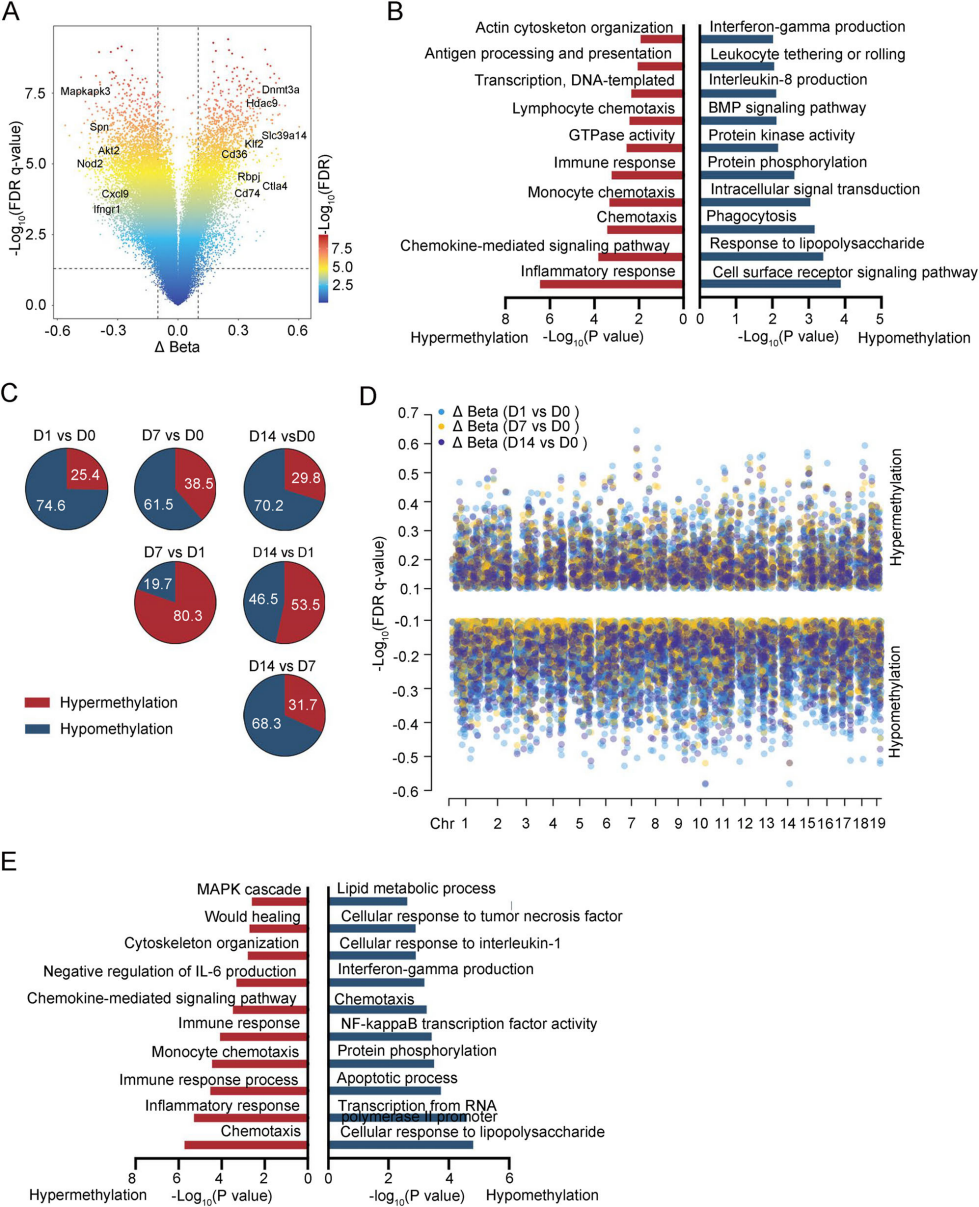
The DMCs in macrophages between BCG‐induced tuberculous pleurisy mouse models and control. (A) Volcano plot showed the DMCs of macrophages in pleural lavage fluid of mice with BCG‐induced tuberculous pleurisy and those in untreated mice. The host genes for the partial differential DMCs are depicted. (B) Pathway enrichment analysis of DMCs of macrophages in pleural lavage fluid of mice with BCG‐induced tuberculosis pleurisy and those in untreated mice. (C) Pie charts showed the status of the DMCs identified in groupwise comparisons. Numbers show the frequency of hyper‐ or hypomethylation. (D) The combined Manhattan plot showed the share DMCs on Days 1, 7, and 14 of infection in BCG‐induced tuberculous pleurisy mouse models compared to Day 0, respectively. Hypermethylated and hypomethylated DMCs were divided into upper and lower sides of the Manhattan plot, respectively. (E) Pathway enrichment analysis of the shared DMCs on Days 1, 7, and 14 of infection in BCG‐induced tuberculous pleurisy mouse models compared to Day 0, respectively.

In macrophages, 13,196 methylation sites differed from uninfected samples. Genes associated with hypomethylation sites were predominantly enriched in interferon‐gamma production, leukocyte tethering or rolling, and phagocytosis signaling pathways. Conversely, genes linked to hypermethylation sites were primarily involved in antigen processing and presentation, immune response, monocyte chemotaxis, chemokine‐mediated signaling pathway, and inflammatory response signaling pathways (Figure 4A,B). Despite these alterations, macrophages exhibited a diminished anti‐inflammatory capacity during sustained BCG treatment. Compared to Day 0, DNA methylation changes on subsequent days were predominantly demethylation (Figure S2B). In contrast, Days 7 and 14 showed mainly hypermethylation compared to Day 1 (Figure 4C). Then, we identified methylation sites that diverged from uninfected samples at Days 1, 7, and 14. A high degree of overlap was observed between the DMCs obtained in different comparisons, with demethylation being the prevailing trend (Figure 4D). These highly shared demethylation sites were primarily concentrated in pathways related to lipid metabolic process, cellular response to tumor necrosis factor, cellular response to interleukin‐1, interferon‐gamma production, chemotaxis, NF‐kappa B transcription factor activity, apoptotic process, and cellular response to lipopolysaccharide (Figure 4E). The above DNA methylation data further corroborated that upon mycobacterium infection, macrophage‐mediated innate immunity responded immediately but subsequently weakened, with this entire process modulated by DNA methylation.

### 2.4 DNA Methylation–Influenced Gene Expression in Macrophages at Different Time Points Post‐BCG Injection

**FIGURE 5:**
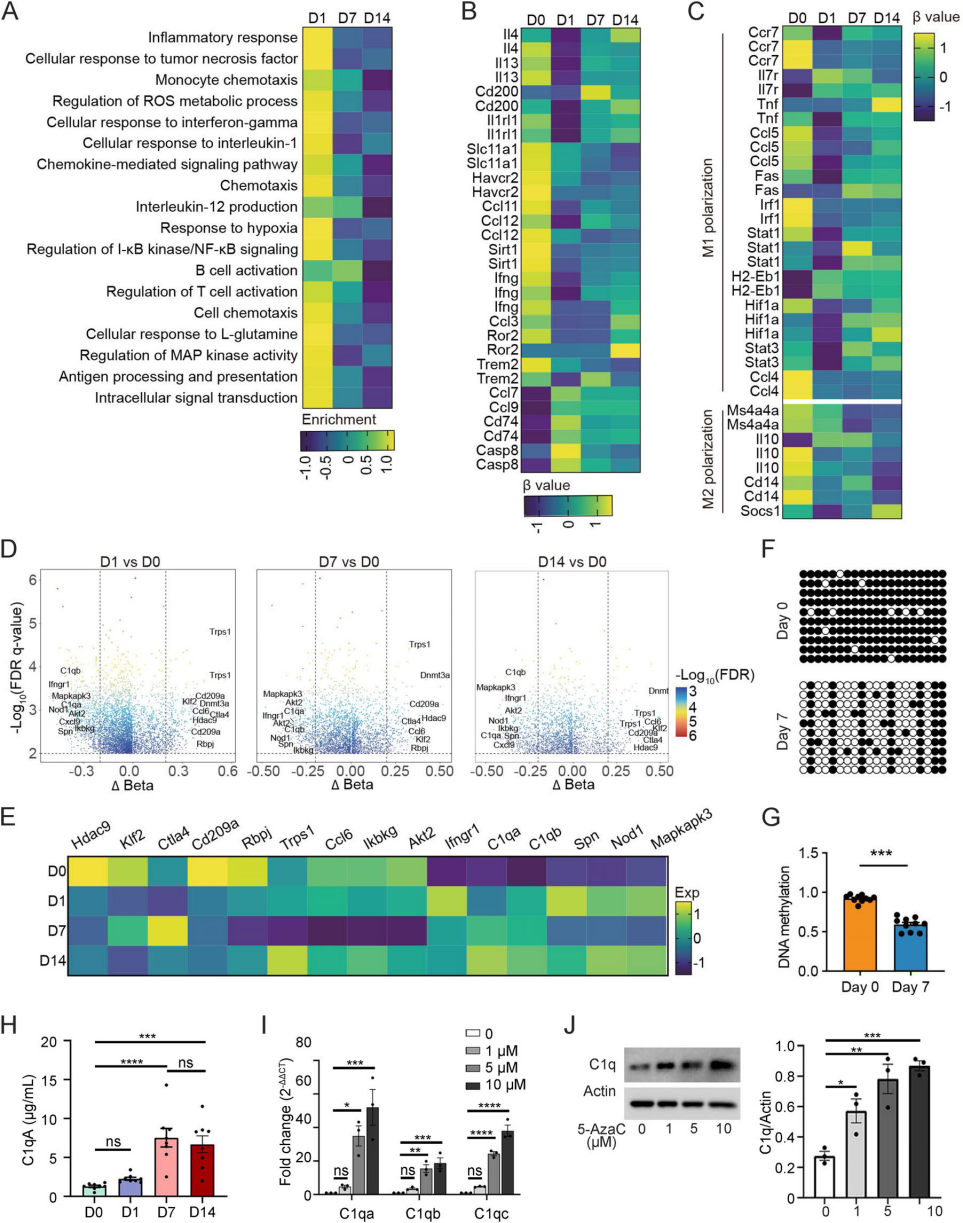
DNA methylation–affected gene expression in macrophages at different times post‐BCG vaccination. (A) Heatmaps showed the differential methylation level of the genes in the corresponding pathways. Each row represents a methylation site. (B) Heatmaps showed the methylation level of the known genes related to macrophage function. Each row represents a methylation site. (C) Heatmaps showed the methylation level of the genes related to macrophage polarization state. Each row represents a methylation site. (D) Volcano plot showed the DMCs between mice infected with BCG for the corresponding number of days and untreated mice. The host genes shared in three groupwise comparisons are labeled. (E) Heatmaps showed the expression level of the corresponding genes. (F) The representative images of BSP assay in macrophages of Days 0 and 7 in the BCG‐induced tuberculous pleurisy mouse model. The black solid circles represent methylated CpG sites, and white hollow circles indicate unmethylated CpG sites. (G) The statistical results of BSP assay in macrophages of Days 0 and 7 in the BCG‐induced tuberculous pleurisy mouse model. (H) Statistical comparisons of the C1qa level in pleural lavage fluid collected from mice at Days 0, 1, 7, and 14 (each *n* = 8). (I) mRNA expression level of C1qa, C1qb, and C1qc in macrophages treated with different concentrations of 5‐azacitidine (each *n* = 3). (J) Protein expression level of C1q in macrophages treated with different concentrations of 5‐azacitidine (5‐AzaC) (each *n* = 3). Data are presented as means ± SEM. *p < 0.05, **p < 0.01, ***p < 0.001, ****p < 0.0001; the comparisons were determined by Student's *t*‐test.

We subsequently assessed the methylation status of established macrophage‐associated functional genes and pathways. The enrichment score of these macrophage pathways linked to anti‐inflammatory function peaked on Day 1 of infection and subsequently decreased gradually (Figure 5A). Notably, CpG sites associated with Il4, Il13, Cd200, and Il1rl1 displayed the lowest methylation levels on Day 1 of infection, followed by an increase over time. Conversely, CpG sites related to Slc11a1, Havcr2, Ccl11, Ccl12, Sirt1, Ifng, Ccl3, Ror2, and Trem2 maintained lower methylation levels throughout the infection compared to noninfected samples. CpG sites linked to Ccl7, Ccl9, Cd74, and Casp8 exhibited the lowest methylation in uninfected samples but increased on Day 1 of infection and then decreased gradually (Figure 5B). Furthermore, most M1 polarization–related genes demonstrated the lowest methylation levels on the first day of infection, followed by a gradual recovery. However, the methylation levels of M2 polarization‐related genes remained relatively stable (Figure 5C), suggesting that macrophages undergo M1 polarization early in infection but cannot sustain it. Consistent with changes in DNA methylation levels, expressions of Il4, Il13, Cd200, and Il1rl1 were highest on the first day of infection and gradually decreased over time. Meanwhile, the expressions of Slc11a1, Havcr2, Ccl12, Sirt1, Ifng, Ccl3, and Trem2 were higher during the whole infection process. The expressions of Ccl7, Ccl9, Cd74, and Casp8 were highest in uninfected samples but decreased on the first day of infection and then gradually increased. Most M1 polarization–related genes demonstrated a higher expression level on the first day of infection, and the expression levels of M2 polarization‐related genes remained relatively stable (Figure S3).

To identify genes with persistently abnormal expression and associated methylation sites during BCG infection, we compared differential methylation sites on Days 1, 7, and 14 to noninfected tissues. Genes involved in methylation sites differing in all three comparisons and ranked high in Δbeta included Hdac9, Klf2, Ctla4, Cd209a, Rbpj, Trps1, and Ccl6, which were hypermethylated in infected tissue. In contrast, Ikbkg, Akt2, Ifngr1, C1qa, C1qb, Spn, IKBKG, Nod1, and Mapkapk3 were demethylated in infected tissue (Figure 5D). RNA sequencing data revealed a distinct pattern of gene expression during infection: Cd209a expression decreased progressively, whereas C1qa and C1qb expression increased consistently (Figure 5E).

Although C1q has been reported to be upregulated in TB patients and was defined as a promising biomarker for TB diagnosis, the underlying mechanism of how C1q expression was regulated during infection was unclear [20, 21]. We isolated the macrophages from the pleural cavity of mice on Days 0 and 7 after BCG injection to perform BSP sequencing and found that the methylation level of macrophage C1qa in pleural lavage fluid on Day 7 after BCG injection was significantly lower than that in control mice (Figure 5F,G), consistent with our DNA methylation chip and RNA sequencing data. Enzyme‐linked immunosorbent assay (ELISA) detection of C1qa in pleural lavage fluid also verified that the expression of C1qa continued to increase with the extension of BCG infection time at the protein level (Figure 5H). To further confirm that changes in C1qa expression were regulated by DNA methylation in macrophages, we treated the macrophages with 5‐azacitidine, a demethylation agent used frequently, at different concentrations, and then analyzed the C1q expression at mRNA and protein levels, respectively. Quantitative polymerase chain reaction (PCR) results suggested that treatment of 5‐azacitidine at 5 and 10 µM could significantly increase C1qa, C1qb, and C1qc mRNA expression; the similar trend was found at 1 µM, although there was no statistical difference (Figure 5I). Consistent with the results from the aspect of mRNA expression, treatment of 5‐azacitidine can also increase the C1q expression at protein level, confirmed by western blot (Figure 5J).

Collectively, these findings highlight the involvement of DNA methylation in macrophage polarization and the regulation of immune‐related gene expression in a mouse model of BCG‐induced tuberculous pleurisy. Notably, the expression of C1q, a key component of the immune response, increased gradually with the duration of infection, emphasizing its significance in the context of macrophage response.

### 2.5 Single‐Cell RNA Sequencing Revealed That C1q^+^ Macrophages Were Enriched in BCG‐Induced Tuberculous Pleurisy Mouse Models

**FIGURE 6:**
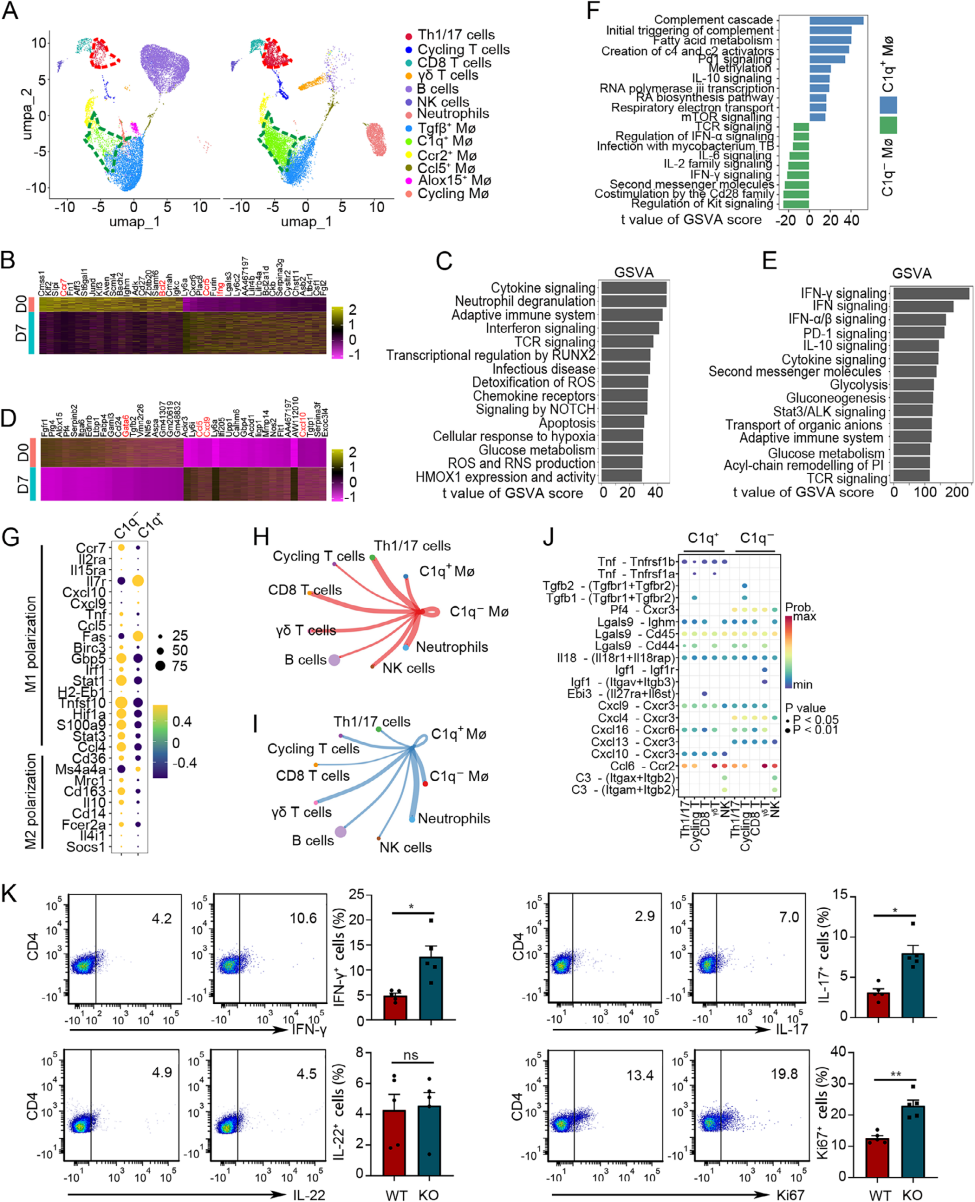
Single‐cell RNA sequencing analysis of pleural lavage fluid of BCG‐induced tuberculous pleurisy mouse models. (A) UMAP plot of cells isolated from pleural lavage fluid colored by the cell types. (B) Heatmaps showed the differential genes in CD4^+^ T cells between Days 7 and 0 in the BCG‐induced tuberculous pleurisy mouse model. (C) The significant differential pathways of CD4^+^ T cells on Day 7 of infection compared to Day 0 in BCG‐induced tuberculous pleurisy mouse model. The pathways were ranked based on *t* value of GSVA score. (D) Heatmaps showed the differential genes in macrophages between Days 7 and 0 in the BCG‐induced tuberculous pleurisy mouse models. (E) The significant differential pathways of macrophages on Day 7 of infection compared to Day 0 in BCG‐induced tuberculous pleurisy mouse models. The pathways were ranked based on *t* value of GSVA score. (F) The significant differential pathways between C1q^+^ and C1q^−^ macrophages on Day 7 of infection in BCG‐induced tuberculous pleurisy mouse models. The pathways were ranked based on *t* value of GSVA score. (G) Dot plot showed the expression levels of the genes related to macrophage polarization state. (H) The number of significant ligand–receptor pairs between C1q^−^ macrophages and other cell types. The edge width is proportional to the number of specified ligand–receptor pairs. (I) The number of significant ligand–receptor pairs between C1q^+^ macrophages and other cell types. The edge width is proportional to the number of specified ligand–receptor pairs. (J) The significant ligand–receptor pairs that contribute to the signaling sending from C1q^+^ or C1q^−^ macrophages to other cell types. The dot color and size represented the calculated communication probability and *p* values. *p* values are computed from one‐sided permutation test. (K) Comparison of Th1, Th17, and Th22 cell populations and Ki67^+^ cell proportions between cells cocultured with macrophages from wild‐type (WT) and C1qa knockout (C1qa^−/−^) mice (each *n* = 5) Data are presented as means ± SEM. *p < 0.05, **p < 0.01; the comparisons were determined by Student's *t*‐test. MΦ, macrophage.

On Day 7 of BCG treatment, both macrophages and CD4^+^ T cells demonstrated robust immune responses. With the development of single‐cell sequencing technology, the function of certain cell subpopulations with specific characteristics attracts more attention. We gradually realized that only a small cell subtype is functional, and different subclusters of the same cell have different functions. For example, what specific function does C1q^+^ macrophage play in the process of BCG infection? To elucidate the gene expression patterns and function of immune cell subclusters in BCG‐induced tuberculous pleurisy mouse models, we extracted immune cells from the pleural lavage fluid 7 days post‐BCG administration, along with cells from untreated mice, for single‐cell transcriptomic sequencing. To minimize individual mouse‐ or sample‐related errors, we pooled pleural lavage fluid specimens from six mice for sequencing. Following raw data processing, filtering of low‐quality genes and cells, and removal of duplicate cells, 26,171 cells were obtained and categorized into 14 cell clusters (Figure S4A–C). Based on the intensity and specificity of cell marker expression, these 14 clusters were classified into 13 cell types, including T cells (marker genes: Cd4, Cd8a, Cd3d, and Cd3e), B cells (marker genes: Ms4a1, Cd79b, Cd19, and Ly6d), NK cells (marker genes: Nkg7, Gzma, Prf1, and Ncr1), macrophages (marker genes: Cd14, Lyz2, and Itgam), and neutrophils (marker genes: S100a8, Rsad2, Isg15, and Il1rn, Figure S4D,E). T cells were further subdivided into four subtypes (Th1/17 cells, CD8 T cells, γδ T cells, and cycling T cells) based on their characteristics, while macrophages were categorized into six subtypes (Tgfβ^+^, C1q^+^, Ccr2^+^, Ccl5^+^, Alox15^+^, and cycling macrophages) according to their marker genes (Figures 6A and S4F–H). Consistent with the aforementioned methylation results, within the Th1/17 cell cluster, we identified that Ifng, along with the cytokine and chemokine receptors Ccr5 and Cxcr6, were significantly upregulated in BCG‐infected compared to uninfected mice. Conversely, the expression of naïve CD4^+^ T cell–related molecules, such as Ccr7 and Bcl2, was downregulated (Figure 6B). Signaling pathways including cytokine signaling, adaptive immune system, interferon signaling, TCR signaling, and chemokine receptors were significantly enriched in macrophages of the *M. tuberculosis* infection environment (Figure 6C). The macrophages in BCG‐infected samples highly expressed Ccl5, Cxcl9, Cxcl10, and other inflammatory factors, while the expression of Gata6, a marker of cavity macrophages, decreased (Figure 6D). In addition to IFN‐γ, cytokine and Stat3/AKT signaling pathways related to anti‐*M. tuberculosis* immune response and glycolysis pathways involved in macrophage M1 polarization, Pd‐1 and IL‐10 pathways associated with immunosuppression were also enriched in infected tissues (Figure 6E).

Surprisingly, Th1/17 cells and C1q^+^ macrophages emerged as the most significantly enriched cell subsets in the BCG infection environment (Figure 6A). Th1/17 cells emerge as the primary adaptive immune cell subsets crucial for anti‐*M. tuberculosis* infection in human TPE [1, 22]. Therefore, we focused on the immune characteristics of C1q^+^ macrophages in BCG‐induced tuberculous pleurisy mouse models. A comparative analysis of functional enrichment pathways between C1q^+^ and C1q^−^ macrophages revealed that inflammatory pathways, including regulation of IFN‐α signaling, IL‐2 family signaling, IFN‐γ signaling, and co‐stimulation by the Cd28 family signaling, were significantly enriched in C1q^−^ macrophages (Figure 6F). This finding correlated with the higher expression of M1 polarization signature genes in C1q^−^ macrophages (Figure 6G). Furthermore, the pathway for *M. tuberculosis* infection was enriched in C1q^−^ macrophages. Notably, BCG infection–associated C1q^+^ macrophages exhibited elevated levels of complement pathway and fatty acid metabolism signaling, similar to the characteristics of infiltrating C1q^+^ macrophages observed in tumor studies. Interestingly, C1q^+^ macrophages also highly expressed IL‐10 and Pd‐1 signaling, two immune response inhibition pathways enriched in BCG‐infected tissues. This suggests that the immunosuppressive function of macrophages during BCG infection might be primarily attributed to C1q^+^ macrophages rather than C1q^−^ macrophages. Methylation, a crucial upstream regulatory factor of C1q expression, was also present in the signaling pathway associated with C1q^+^ macrophages.

To examine the potentially altered interactions between C1q^+^ and C1q^−^ macrophages and other immune cell subpopulations, we utilized the scRNA‐seq data to predict the cellular communication of these two cell types using the CellChat package (Figure S5A). Our analysis focused on the changes in intercellular communication between macrophages, T cells, and NK cells. Notably, we observed that the interaction of C1q^−^ macrophages with T cells and NK cells was mediated by the Tnf‐Tnfrsf1b ligand–receptor pairs. Conversely, Cxcl4/Cxcl13‐Cxcr3 and its downstream pathway related to immune response suppression were more prominent in C1q^+^ macrophages (Figures 6H–J and S4B–F). The TNF‐associated DNA methylation changes detected in T cells using DNA methylation microarrays in Figure 2 may be attributed to these ligand–receptor interactions with T cells. During BCG vaccine–induced tuberculous pleural infection, modifications in intercellular communication events might modulate downstream cell signaling pathways and influence DNA methylation profiles in CD4^+^ T cells by enhancing or reducing the ligand and receptor roles involved in inflammatory cytokines or immunosuppression, thereby altering the expression of key genes in T cells and regulating the cell immunophenotype. To verify the regulatory role of macrophages on T cell function under BCG infection, we first isolated macrophages from pleural cavity of wild‐type (WT) and C1qa knockout mice at Day 7 post‐BCG injection and cultured with naïve CD4^+^ T cells. Three days later, we collected the cells and analyzed the Th cell differentiation and CD4^+^ T cell proliferation by flow cytometry (Figure 6K). The results demonstrated that there were more differentiated Th1 and Th17 cells when cocultured with C1qa knockout macrophages compared to WT ones. Besides, the proportion of proliferated CD4^+^ T cells was higher when cocultured with C1qa knockout macrophages compared to WT ones. Interestingly, there was no obvious difference for Th22 cells between two groups. Our findings suggest that C1q^−^ macrophages exhibit a stronger anti‐infection immune function, whereas C1q^+^ macrophages tend to display immunosuppressive properties.

### 2.6 *M. tuberculosis* Infection Induces C1q Expression and Displays an Inhibitory Phenotype

**FIGURE 7:**
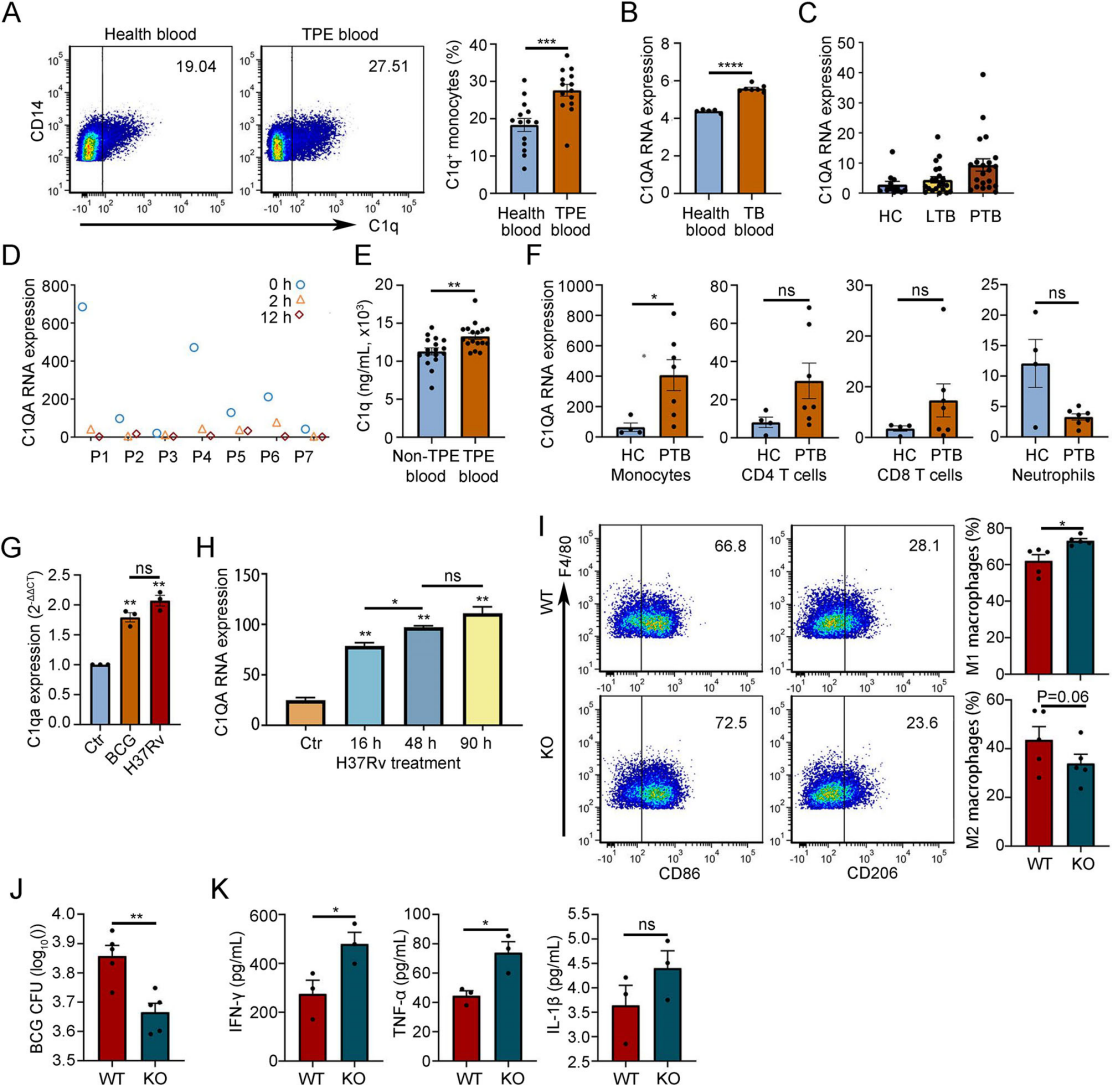
C1q promoted the development of tuberculosis (TB). (A) Proportion of C1q^+^ monocytes in peripheral blood of TPE patients and healthy person. (B) Expression level of C1QA in PBMC of TB patients and healthy person. The data were collected from GEO database (GSE119143). (C) Expression level of C1Q in peripheral blood of TB patients. The data were collected from GEO database (GSE19444). (D) Expression level of C1QA in PBMCs of the same patient at diagnosis of TB (0 month), 2 months after treatment (2 months), and 6 months after treatment (6 months). The data were collected from GEO database (GSE19491). (E) Serum C1q levels in patients with TPE or non‐TPE. (F) Expression level of corresponding cell derived C1QA in peripheral blood of patients with TB. The data were collected from GEO database (GSE19491). (G) Quantitative PCR to detect the expression of C1qa in monocytes isolated from mouse blood treated with protein lysates of BCG or H37RV. (H) The expression level of C1Q in THP‐1 cells treated with H37Rv at different times. The data were collected from GEO database (GSE19052). (I) Comparison of proportions of the M1 and M2 macrophages in pleural lavage fluid of wild‐type (WT) and C1qa knockout (C1qa^−/−^) mice (each *n* = 5). (J) Comparisons of the BCG bacterial load (each *n* = 5) between C1qa^−/−^ and WT mice with BCG‐induced tuberculous pleurisy at Day 7. (K) Comparisons of the level of IFN‐γ, TNF‐α, and IL‐1β (each *n* = 3) between C1qa^−/−^ and WT mice with BCG‐induced tuberculous pleurisy at Day 7. Data are presented as means ± SEM. *p < 0.05, **p < 0.01, ***p < 0.001; the comparisons were determined by Student's *t*‐test. ATB, active tuberculosis; Ctr, control; LTB, latent tuberculosis.

To examine the clinical relevance of C1q and C1q^+^ macrophages in TPE, we isolated CD14^+^ monocytes from PBMC of TPE patients and healthy controls (HCs). Flow cytometry analysis revealed an increased proportion of C1q^+^ monocytes in TPE patients (Figure 7A). Corroborating this finding, an analysis of the GEO dataset (GSE119143 and GSE19444) demonstrated elevated C1Q mRNA expression in PBMC of TB patients compared to HCs, with higher expression levels observed in patients with active TB versus latent TB (Figure 7B,C). Berry et al. conducted a microarray analysis of PBMC samples from seven TB patients at diagnosis, 2 months, and 12 months post anti‐TB treatment [23]. Our analysis of their data indicated a significant decrease in C1Q mRNA expression levels after treatment, with C1Q expression becoming virtually undetectable in six patients after 12 months of treatment (Figure 7D). In line with previous studies, we observed higher levels of C1q in pleural effusion of TPE patients compared to non‐TPE patients (Figure 7E) [24]. To further investigate C1q expression in different immune cell types, we evaluated PBMC expression profiles from various immune cells in TB patients and HCs. Among these cells, only monocytes exhibited increased C1q expression, while CD4^+^ T cells, CD8^+^ T cells, and neutrophils did not display significant changes in C1q expression levels (Figure 7F).

To evaluate the effect of mycobacterial infection on C1q expression, we isolated monocytes from mouse blood and treated them with protein lysates of BCG and H37Rv, respectively. Quantitative PCR analysis revealed that protein lysates of both BCG and H37Rv stimulated C1qa expression in monocytes (Figure 7G). Kumar et al. stimulated THP‐1 cells, a model for human monocytes [25], with H37Rv and conducted expression profile microarray detection [26]. Consistent with the results from mouse monocytes, we observed that the expression level of C1Q mRNA in THP‐1 cells gradually increased after H37Rv treatment (Figure 7H). To test the effects of C1q in vivo, we established mouse pleurisy models using WT and C1qa knockout mice and collected pleural lavage fluid (flushed with 1 mL saline) to analyze the macrophage subpopulations, BCG burden, and related inflammatory factors. The results showed that knockout of C1qa gene increased proportion of M1 macrophages in pleural cavity, decreased the CFU burden of BCG, and increased levels of anti‐*M. tuberculosis* immune–related cytokines like IFN‐γ and TNF‐α (Figure 7I–K). Collectively, these findings suggest a potential role for C1q in macrophage‐mediated immunity against TB.

## 3 Discussion

TB remains a leading cause of mortality from a single infectious agent, second only to COVID‐19. The limited protective efficacy of vaccines in adults contributes significantly to the high incidence of TB despite the cooperative efforts of innate and adaptive immune responses in combating the disease. Notably, 5%–10% of infected individuals progress to active TB. DNA methylation plays a crucial role in regulating immune‐related gene expression and functions in anti‐*M. tuberculosis* immune regulation [27]. However, the mechanisms by which DNA methylation regulates macrophage and CD4^+^ T cell responses during the progression of infection in mouse models remain unclear.

In this study, we conducted a comprehensive analysis of the immune response during BCG‐induced pleurisy in mice, focusing on DNA methylation patterns and RNA sequencing data from macrophages and CD4^+^ T cells isolated from pleural lavage fluid at specific time points (Days 0, 1, 7, and 14). Our findings provided valuable insights into the dynamic interaction between these immune cells during the progression of infection. In the BCG‐induced pleurisy model, we observed a distinct pattern in the population dynamics of macrophages and CD4^+^ T cells. Notably, there was a significant increase in macrophage counts on Day 1 post‐BCG injection, followed by a gradual decrease to a relatively lower level. Conversely, the number of CD4^+^ T lymphocytes gradually increased as the infection progressed. These observations align with previous reports, indicating that macrophages are the primary responders during the early stages of infection, while CD4^+^ T cells play a more significant role in later stages [4, 28]. Our DNA methylation analysis of macrophages and CD4^+^ T cells revealed that the highest level of hypomethylation in macrophages occurred at Day 1 post‐BCG injection, indicating a significant activation of the demethylation state that promotes gene expression activity. Subsequently, the hypomethylation level decreased at Days 7 and 14. In contrast, the DNA hypomethylation level in CD4^+^ T cells gradually increased during the infection. These findings suggest that while macrophages undergo rapid epigenetic reprogramming early in infection, the epigenetic changes in CD4^+^ T cells occur more gradually over time. To further corroborate our findings, we analyzed the correlation of DNA methylation between Day 0 and the other time points. The results demonstrated that the correlation coefficient was smallest on Day 1 in macrophages, indicating significant epigenetic changes at this early time point. Conversely, the correlation coefficient decreased with time in CD4^+^ T cells, suggesting a more gradual accumulation of epigenetic modifications. Our findings were consistent with the established understanding that innate immune cells like macrophages are the predominant cell populations combating *M. tuberculosis* at the early stage of infection, whereas the establishment of effective adaptive immunity requires 2 weeks or longer [5, 9, 29, 30, 31].

Research has extensively documented that cytokines such as IFN‐γ, IL‐2, TNF‐α, and IL‐17 play crucial roles in combating *M. tuberculosis* and in BCG‐induced protection against TB [32, 33, 34]. Our analysis of DNA methylation status in macrophages and CD4^+^ T cells following BCG challenge revealed that the hypomethylation level of IFN‐γ was significantly upregulated in both cell populations. IL‐2 upregulation was primarily observed in CD4^+^ T cells, while TNF‐α upregulation was mainly seen in macrophages (Figures 2B,E and 4B,E). These findings demonstrate that DNA methylation regulates IFN‐γ production in both macrophages and CD4^+^ T cells, while it specifically affects IL‐2 production in CD4^+^ T cells and TNF‐α production in macrophages. The methylation and demethylation of genomic DNA emerge as critical epigenetic factors governing this process.

Following *M. tuberculosis* stimulation, CD4^+^ T cells exhibit increased activation, proliferation, and production of IFN‐γ, IL‐2, and TNF‐α [35]. The differentiation of naïve CD4^+^ T cells into specific Th cell subsets is governed by various combinations of transcription factors and cytokines [1]. Previous research, including our own, has extensively demonstrated the predominance and vital roles of Th1, Th17, Th22, and Th9 cells in the pathogenesis of TPE [1, 36]. In the present study, we observed that compared to the naïve state at Day 0, CD4^+^ T cells from Days 1, 7, and 14 displayed progressively enhanced IFN‐γ responses, activation, proliferation, and differentiation (Figure 3A). Further analysis of DNA methylation status of critical genes governing Th cell differentiation revealed that the degree of DNA hypomethylation in key genes regulating the differentiation of Th1, Th17, and Th22 cells generally increased over time following BCG challenge (Figure 3B). Conversely, while some genes regulating Th9 differentiation showed increased DNA hypomethylation, others did not (Figure 3C). This observation suggests that upon *Mycobacterium bovis* infection, the differentiation of Th1, Th17, and Th22 cells is regulated by DNA methylation status, whereas DNA methylation may not be a prominent regulatory factor for Th9 differentiation.

Complement C1q has been identified as upregulated in active TB patients and recognized as a promising biomarker for TB diagnosis [20, 21, 24, 37]. C1q is an 18‐subunit–organized protein assembled by three different subunits (C1qA‐C1qC‐C1qB) at a 1:1:1 ratio. The three corresponding genes are closely bundled on chromosome 1, and their expressions are synchronously regulated [38], although the underlying mechanism of upregulation in active TB remains unknown. In our analysis of DNA methylation profiles following BCG injection, we observed that the methylation levels of both C1qa and C1qb genes were decreased (Figure 5D), suggesting that their expressions were upregulated by demethylation as at least one important mechanism. Consistent with the methylation data, the RNA‐seq results also demonstrated that C1q gene expressions were upregulated following BCG injection in vivo (Figure 5E) and BCG lysate induction in vitro (Figure 7G).

In our single‐cell sequencing analysis of immune cells within pleural lavage fluid, we observed that C1q^+^ macrophages constituted a relatively distinct subpopulation with a marked increase in number following BCG injection (Figure 6A). GSVA analysis revealed that C1q^+^ macrophages exhibited inhibitory characteristics, with upregulation of PD‐1 and IL‐10 signaling (Figure 6F). Cell‐to‐cell communication analysis demonstrated that C1q^+^ macrophages had fewer interactions with other effector cells and might exert their inhibitory effects on T cells through Cxcl4, Cxcl13, and their corresponding receptors (Figure 6H–J). Our in vivo study using a mouse model showed that knockout of the C1qa gene decreased the CFU burden of BCG and increased levels of anti‐*M. tuberculosis* immune–related cytokines such as IFN‐γ and TNF‐α (Figure 7J,K). These findings support the hypothesis that drugs targeting C1q blockade may be promising candidates for TB treatment. An anti‐C1q antibody, ANX005, developed by biotech startup Annexon, has entered Phase 2 clinical trials in patients with Huntington's disease, with participants' clinical status remaining stable over 6 months of dosing and 3 months of follow‐up. Further clinical research is needed to determine whether anti‐C1q antibodies can be used to treat TB or enhance the immune effect of the BCG vaccine. Additionally, while BCG is a vaccine used to prevent TB in humans, it is less effective against TB in adults [9]. The potential role of these immunosuppressive C1q^+^ macrophages in the poor protective effect of the BCG vaccine is an intriguing hypothesis that warrants further exploration and verification in clinical studies.

Our study has certain limitations. We primarily focused on macrophages and CD4^+^ cells in our DNA methylation and RNA expression analysis, excluding other cell populations such as neutrophils and B cells. In our single‐cell sequencing analysis, we observed a significant increase in neutrophils post‐BCG infection, aligning with previous reports of pronounced neutrophil accumulation in the pleural cavity 24 h after BCG injection [3]. This might represent an acute inflammatory response to BCG injection. We also noted a substantial decrease in B cells post‐BCG injection, consistent with findings from a study on group B streptococci‐induced mouse peritonitis [39]. Given the fixed total number of cells analyzable by single‐cell sequencing, we hypothesize that the influx of neutrophils, macrophages, T cells, and other immune cells following BCG injection reduced the proportion of B cells, resulting in fewer captured B cells. Due to the scarcity of neutrophils at Day 0 and B cells at Day 7, and their relatively minor contribution to the anti‐*M. tuberculosis* immune response compared to CD4^+^ T cells and macrophages, we did not conduct further analysis of these cells in this study. However, recognizing their roles in humoral and innate immune responses, our future research will investigate their impact on TB and TPE.

## 4 Materials and Methods

### 4.1 Animals and Pleurisy Mouse Model

Six‐ to eight‐week‐old C57BL6 mice were utilized in pleurisy model. WT mice were purchased from Beijing Vital River Laboratory Animal Technology Co. Ltd., while the C1qa knockout mice were generously provided from Chao Xiong (Fuwai Hospital, Chinese Academy of Medical Sciences and Peking Union Medical College). The pleurisy mouse model was established and modified based on previous studies [3, 4].

### 4.2 Methylation Analysis

The TIANamp Genomic DNA Kit (TIANGEN, China) was used to extract and purify genomic DNA from cells. Samples underwent bisulfite conversion utilizing the EZ DNA Methylation‐Gold Kit (Zymo Research, CA, USA) before being hybridized on Infinium Mouse Methylation BeadChip (Illumina Inc., San Diego, CA, USA).

### 4.3 RNA‐seq Sample Processing and Analysis

RNA sequencing was performed on a batch of samples to evaluate methylation patterns. Total RNA was extracted using TRIzol (Invitrogen) according to the manufacturer's instructions. The strand‐specific libraries were generated with NEBNext UltraTM RNA Library Prep Kit for Illumina (NEB, Ipswich, MA, USA) following the manufacturer's recommendations and sequenced on the Illumina NovaSeq 6000 System. The paired‐end reading of 150 bp was produced and subsequently mapped to GRCm39 reference genome. Differentially expressed genes were identified using the Limma (v.3.54.2) package.

### 4.4 Bisulfite Sequencing PCR

Macrophages were isolated from the pleural cavity of WT mice at Day 0 (prior to BCG injection) and Day 7 (following BCG injection), and genomic DNA was extracted. When treated with bisulfite, all unmethylated cytosines are converted to uracil, while methylated cytosines remain unchanged. Primers were designed flanking the CpG island of C1qa for PCR, and the resulting target product was purified for TA cloning. Ten positive clones were selected from each set and subsequently sequenced. Finally, the obtained sequences were compared to the original sequences to identify and quantify methylation sites and their respective numbers.

### 4.5 Statistics

Data are presented as means ± SEM. Statistical comparisons between groups were performed with Student's *t*‐test. Statistical calculations were analyzed by R software. *p* < 0.05 was considered statistically significant.

More detailed materials and methods information is available in Supporting Information.

## Author Contributions

M.‐M.S. and F.‐S.Y. carried out most of the wet and dry experiments and analyzed the data. Q.‐Y.C. and X.Z. performed the mouse model–related experiments. S.‐F.D. and R.‐Q.W. collected samples and performed related experiments. M.‐M.S. and F.‐S.Y. drafted the manuscript. F.‐S.Y. and H.‐Z.S. revised the manuscript, conceived the idea, and supervised the project. All authors read and approved the final version of the manuscripts.

## Ethics Statement

The animal experiments were approved by the Animal Welfare & Ethics Committee of Capital Medical University (No. AEEI‐2021‐193). The clinical sample–related study was approved by the Ethics Committee of Beijing Chaoyang Hospital, Capital Medical University (No. 2021‐ke‐9).

## Conflicts of Interest

The authors declare no conflicts of interest.

## Supporting information



Supporting Information

## Data Availability

The data presented in this study are available from the corresponding author upon reasonable request. The raw sequence data reported in this paper have been deposited in the Genome Sequence Archive (Genomics, Proteomics & Bioinformatics 2021) in National Genomics Data Center (Nucleic Acids Res 2022) and China National Center for Bioinformation/Beijing Institute of Genomics, Chinese Academy of Sciences (CRA014365 and OMIX005631) that are publicly accessible at https://ngdc.cncb.ac.cn.
