# Supplementary material for: Dynamic Alterations in DNA Methylation of CD4+ T Cells and Macrophages in a Murine Model of Tuberculous Pleural Infection Induced by BCG Vaccination
Source: MedComm (2020). 2025 Apr 1;6(4):e70166. doi: 10.1002/mco2.70166 (PMC11959155; doi:10.1002/mco2.70166)
Supplement: Supplementary file 1 — Supporting Information [file MCO2-6-e70166-s001.pdf]

Dynamic Alterations in DNA Methylation of CD4$^+$ T Cells and Macrophages in a Murine Model of Tuberculous Pleural Infection Induced by BCG Vaccination

Running title: DNA methylation in BCG vaccine-induced pleurisy

Ming-Ming Shao[1,2], Qing-Yu Chen[1], Xin Zhang[1], Shu-Feng Dong[1], Rui-Qi Wei[1], Huan-Zhong Shi[1,*], Feng-Shuang Yi[1,2,*]

[1]Department of Respiratory and Critical Care Medicine, Beijing Institute of Respiratory Medicine and Beijing Chao-Yang Hospital, Capital Medical University, Beijing 100020, China.

[2]Medical Research Center, Beijing Institute of Respiratory Medicine and Beijing Chao-Yang Hospital, Capital Medical University, Beijing 100020, China.

*Correspondence to:

Feng-Shuang Yi, yifengshuang@ccmu.edu.cn

Huan-Zhong Shi, shihuanzhong@sina.com

Materials and Methods

Animals and pleurisy mouse model

Prior to establishing pleurisy, mice received a subcutaneous injection of 1×10^6 attenuated *M. bovis* (BCG Shanghai strain) in a volume of 0.1 mL. Two weeks later, an additional dose of 5×10^6 attenuated *M. bovis* per 0.1 mL was injected into the pleural cavity of the mice. Mice were sacrificed at day 1, day 7 and day 14 after injection, and lung tissues and pleural lavage fluid (with 1ml saline) were subsequently collected. The animal experiments were approved by the Animal Welfare & Ethics Committee of Capital Medical University (No. AEEI-2021-193).

Hematoxylin and eosin (H&E) staining and Acid Fast Bacteria (AFB) staining

Tissues underwent stepwise dehydration using ethanol solutions with varying concentrations, followed by transparency treatment with xylene before being embedded in paraffin wax. Sections measuring 4 μm in thickness were prepared for both H&E staining and AFB staining respectively. Prior to staining, paraffin sections were dewaxed accordingly. Hematoxylin and eosin served as stains for H&E analysis, while carbolic acid combined with methylene blue was employed for AFB staining.

Cell sorting and flow cytometry

Pleural lavage fluid was centrifugated at 400g for 10 min to separate the supernatant and cell pellet; following red blood cell lysis, cells were stained with specific antibodies for flow cytometry analysis. The fluorescent antibodies used for flow cytometry analysis of mouse sample includes anti-CD45, -CD4, -F4/80, -CD11b, -CD86, -CD206 (Thermo Fisher Scientific, Waltham, MA,USA). $CD4^+$ T cells and $F4/80^+$ macrophages were isolated by BD Arial II., these isolated cells were then separated into two parts for subsequent DNA methylation analysis and RNA sequencing. To determine the

differentiation and proliferation of Th cells, anti-CD3, -CD4, -IFN-γ, -IL17, -IL-22, -Ki67 antibodies were used.

Human peripheral blood and TPE samples were centrifugated to obtain the cell pellet, the supernatant was stored at -80°C for subsequent enzyme-linked immunosorbent assay (ELISA) measurement. Mononuclear cells were isolated from peripheral blood with Ficoll solution and stained with specific antibodies for flow cytometry analysis. The fluorescent antibodies for flow cytometry analysis of human sample includes anti-CD45, -CD14, -C1QA (Thermo Fisher Scientific). Human pleural effusion and blood were obtained from the Department of Respiratory and Critical Care Medicine, Beijing Chaoyang Hospital, Capital Medical University. This study was approved by the Ethics Committee of Beijing Chaoyang Hospital, Capital Medical University (No. 2021-ke-9), and all subjects had written informed consent.

Methylation analysis

The TIANamp Genomic DNA Kit (TIANGEN, China) was used to extract and purify genomic DNA from cells. The purified DNA was quantified using Qubit (Invitrogen, Carlsbad, CA, USA) according to the manufacturer's instructions. Samples underwent bisulfite conversion utilizing the EZ DNA Methylation-Gold™ Kit (Zymo Research, CA, USA) before being hybridized on arrays as per manufacturer guidelines. The Infinium Mouse Methylation BeadChip (Illumina, Inc., San Diego, CA, USA), the array detecting more than 285,000 methylation sites per sample at single nucleotide resolution was used to analyze DNA methylation in mice. GenomeStudio Software 2011.1 (Illumina) facilitated evaluation and analysis of raw signal intensities. Beta values representing DNA methylation levels were derived from original IDAT files and normalized within GenomeStudio by referencing probes while subtracting background signals. To ensure data

reliability and stability, probes that did not meet quality criteria, including those with P values > 0.01, manufacturing flagged (MFG) probes, XY chromosome probes, and genotyping probes, were excluded from the consideration. IDAT files were then processed using the SeSAMe and SeSAMeStr packages for comprehensive analysis. Differential DNA methylation was determined using a cutoff of P value < 0.05 and a Δ beta > 0.1.

RNA preparation and quantitative real-time polymerase chain reaction analysis (qRT-PCR)

Total RNA extraction was conducted using TRIzol according to manufacturer guidelines. Reverse transcription was carried out employing the PrimeScriptTM RT Reagent Kit with gDNA Eraser (TaKaRa, Japan). Quantitative reverse transcription PCR (qRT-PCR) was performed on an ABI Prism 7900 system (Applied Biosystems, CA, USA) utilizing SYBR Green (TaKaRa) methodology with gene-specific primers included in each reaction setup. The measurement of RNA expression levels was normalized against GAPDH transcript levels across three independent transfection experiments; each experiment included at least three replicates.

Cell culture

To co culture of macrophages and naïve $CD4^+$ T cells, macrophages were isolated from pleurisy mouse pleural cavity of both WT and C1qa knockout mice at day 7, and cultured in RPMI Medium 1640 (Solarbio) containing 10% heat-inactivated FBS, 100 U/ml Penicillin, 100 mg/ml Streptomycin, and 20 ng/mL macrophage colony-stimulating factor (M-CSF) in 5% CO_2 at 37°C. Naïve $CD4^+$ T cells were isolated from spleen of naïve WT mice using Mouse Naïve $CD4^+$ T Cell Isolation Kit accompanied with LS Separation colums (Miltenyi Biotec). The naïve $CD4^+$ T cells were cultured in the presence of 2 μg/mL anti-CD3 antibody and 0.5 μg/mL anti-CD28 antibody overnight before co cultured with macrophages. The activated naïve $CD4^+$ T cells were collected,

counted, and then were co cultured with WT and C1qa knockout macrophages, respectively, in the presence of M-CSF. Three days later, Th cell differentiation and proliferation were analyzed by flow cytometry.

To verify the effects of DNA methylation on C1q expression, different concentrations (0, 1, 5, 10 μM) of 5-Azacitidine (MedChemExpress) were treated to macrophages isolated from pleural cavity of WT mice. two days later, cells were collected for qPCR and western blot analysis, respectively.

Western blot

After quantification, samples were subjected to SDS-PAGE, and then were transferred onto PVDF membranes. After blocking with 5% nonfat milk, specific primary Abs to C1q (Abcam) were used to probe the target proteins. After incubating with HRP-conjugated secondary Abs, the ECL system (MilliporeSigma) was used to detect the bands. Actin served as an internal control. To detect its expression, we used strip buffer to recover the membranes, and proceeded the similar procedure of C1q. ImageJ software was used to quantify the relative expression.

Single cell sequencing sample processing and analysis

Pleural lavage fluid samples were collected from BCG-induced tuberculous pleurisy mouse models on day 0 and day 7 of infection. Cells were collected by centrifugation and single-cell sequencing was performed. The process was briefly described as follows. Pleural lavage fluid was centrifugated at 400g for 10 min to obtain the cell pellet, after red blood cell lysis, the cells were resuspended with PBS for further analysis. Single cells were captured using GEXSCOPE® (Singleron, China) microfluidic chip and barcoding beads with unique cell barcode were added to the chip microhole to ensure that only one barcoding beads fallen into each microhole. After cell lysis, barcoding beads with unique molecular identifiers (UMI) captured mRNA by binding to poly (A) tail on mRNA and

labeled the cell and mRNA. Barcoding beads in the chip were collected, and the mRNA captured by barcoding beads was reverse-transcribed into cDNA and amplified. A sequencing library suitable for illumina sequencing platform was constructed after the cDNA was segmented and spliced, and then sequenced using a NovaSeq 6000 System (Illumina) with 150-bp pair-end reading strategy. The generated single-cell sequencing data was processed using CeleScope (Singleron). Reads were aligned to the GRCm39 reference genome. The results of the CeleScope analysis contained the counted values of UMIs assigned to each gene, for each cell of each individual samples, using all mapped reads. The package Seurat (v.4.3.0) based on R software (v.4.0.2) was used to create data analysis objects. For a single sample, data quality control analysis was first mainly performed: 1. the cells that expressed less than 200 genes or more than 5000 genes; 2. the cells that expressed more than 10% of mitochondrial genes; 3. the genes detected in less than 200 cells were all filtered out. DoubletFinder (v.2.0.1) was used to identify and exclude cell doublets. Next, we used the IntegrateData function in Seurat to consolidate data and remove batch effects, the NormalizeData function to logarithmically transform the data, the FindVariableGenes function to identify the highly variable genes, the ScaleData and RunPCA functions to scale the dataset, and the RunUMAP functions to nonlinear dimensionality reduction of data. We annotated the cell types on the the result of SingleR (v.2.0.0) analysis and the canonical marker genes (T cells: Cd4, Cd8a, Cd3d and Cd3e; B cells: Ms4a1, Cd79b, Cd19 and Ly6d; NK cells: Nkg7, Gzma, Prf1 and Ncr1; Macrophages: Cd14, Lyz2 and Itgam; Neutrophils: S100a8, Rsad2, Isg15 and Il1rn).

Cell-to-cell interaction analysis

As described by Jin et.al [1], we used CellChat (v.0.5.5) for cell-to-cell interaction analysis in R platform. The gene expression data from cells served as input to model the probability of

intercellular communication by integrating prior knowledge regarding interactions between gene expression and signaling ligands, receptors, and their cofactors. A P-value threshold of < 0.05 was considered statistically significant.

ELISA

The concentration of C1q protein in pleural fluid samples was quantified using a C1q enzyme-linked immunosorbent assay kit (Thermo Fisher Scientific), adhering strictly to manufacturer protocols for accurate results interpretation. TPE diagnosis relied upon positive findings from either pleural fluid or biopsy specimens via Ziehl-Neelsen staining or Mycobacterium tuberculosis culture; alternatively, granuloma presence in pleural biopsy specimens also indicated TPE diagnosis criteria fulfillment. Non-TPE conditions encompassed malignant pleural effusion as well as effusions associated with bacterial pneumonia, lung abscesses, and bronchiectasis infections. To determine the concentration of C1q in mouse pleural lavage fluid (flushed with 1ml saline), Mouse Complement C1q Subcomponent Subunit A (C1QA) ELISA Kit was used (CSB-EL003637MO, CUSABIO) according to the manufacturer's instructions.

References

1. Jin S, Guerrero-Juarez CF, Zhang L, et al. Inference and analysis of cell-cell communication using CellChat. *Nat Commun.* 2021;12(1):1088.

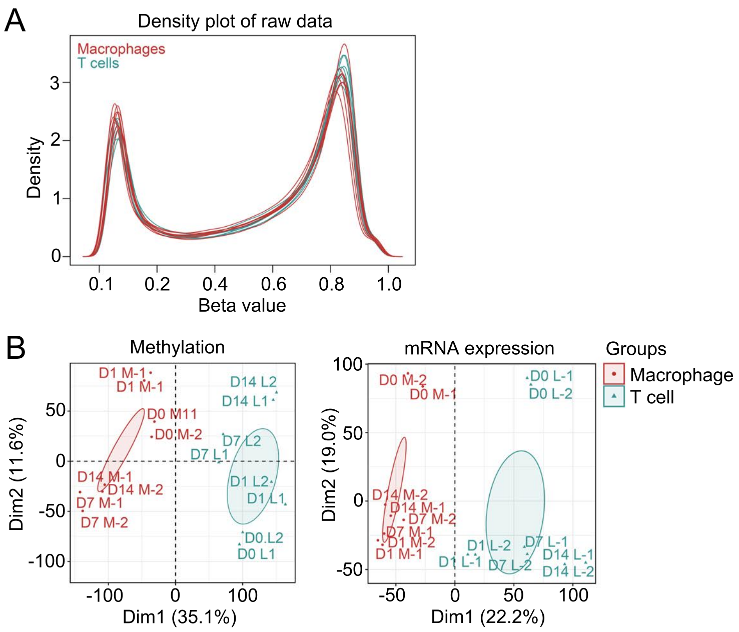

Figure S1. Quality control of Infinium mouse methylation array data.

(A) The distribution of methylation row data of 16 immune cell samples collected from pleural lavage fluid of BCG induced tuberculous pleurisy models. (B) Principal component analysis (PCA) of DNA methylation levels (left panel) and mRNA levels (right panel) are displayed as PCA plot.

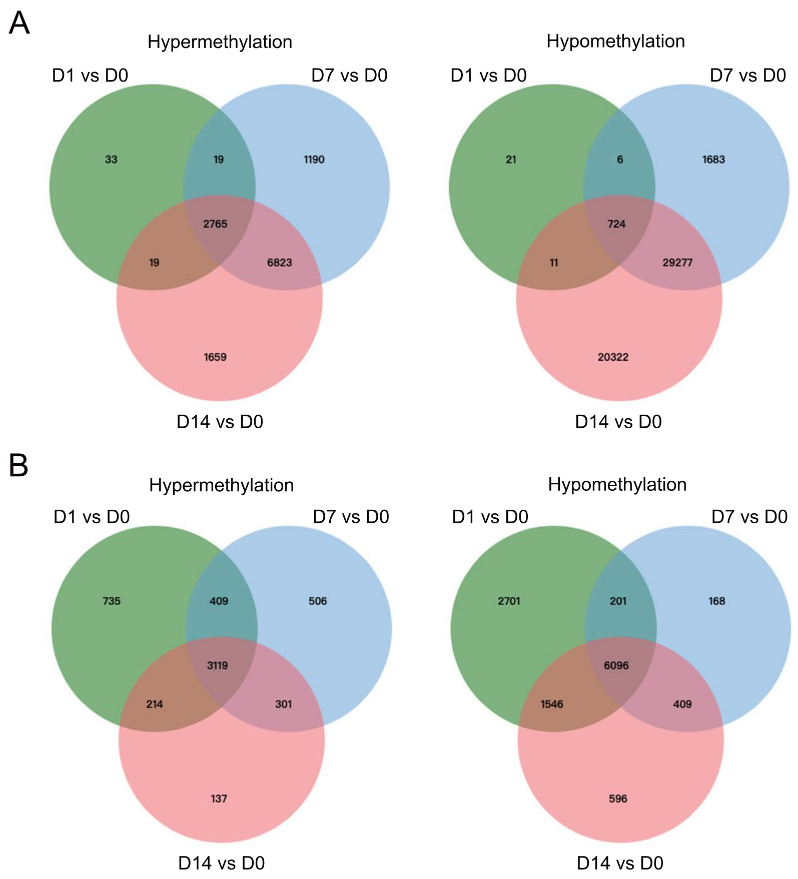

Figure S2. The Venn diagram of differential methylation sites.

(A, B) Venn diagram of hyper- (left panel) or hypo- (right panel) methylated genes in the different stages of BCG-induced tuberculous pleurisy model mice compared to untreated mice. A was the Venn diagram of macrophages and B was the Venn diagram of $CD4^{+}$ T cells.

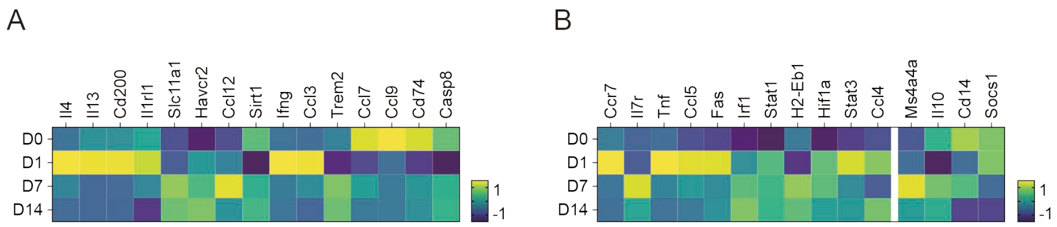

Figure S3. The RNA expression of immune regulation genes and polarization makers in macrophages.

(A) Heatmap showed the expression of macrophage immune regulation genes. (B) Heatmap showed the expression of the genes involved in macrophage polarization.

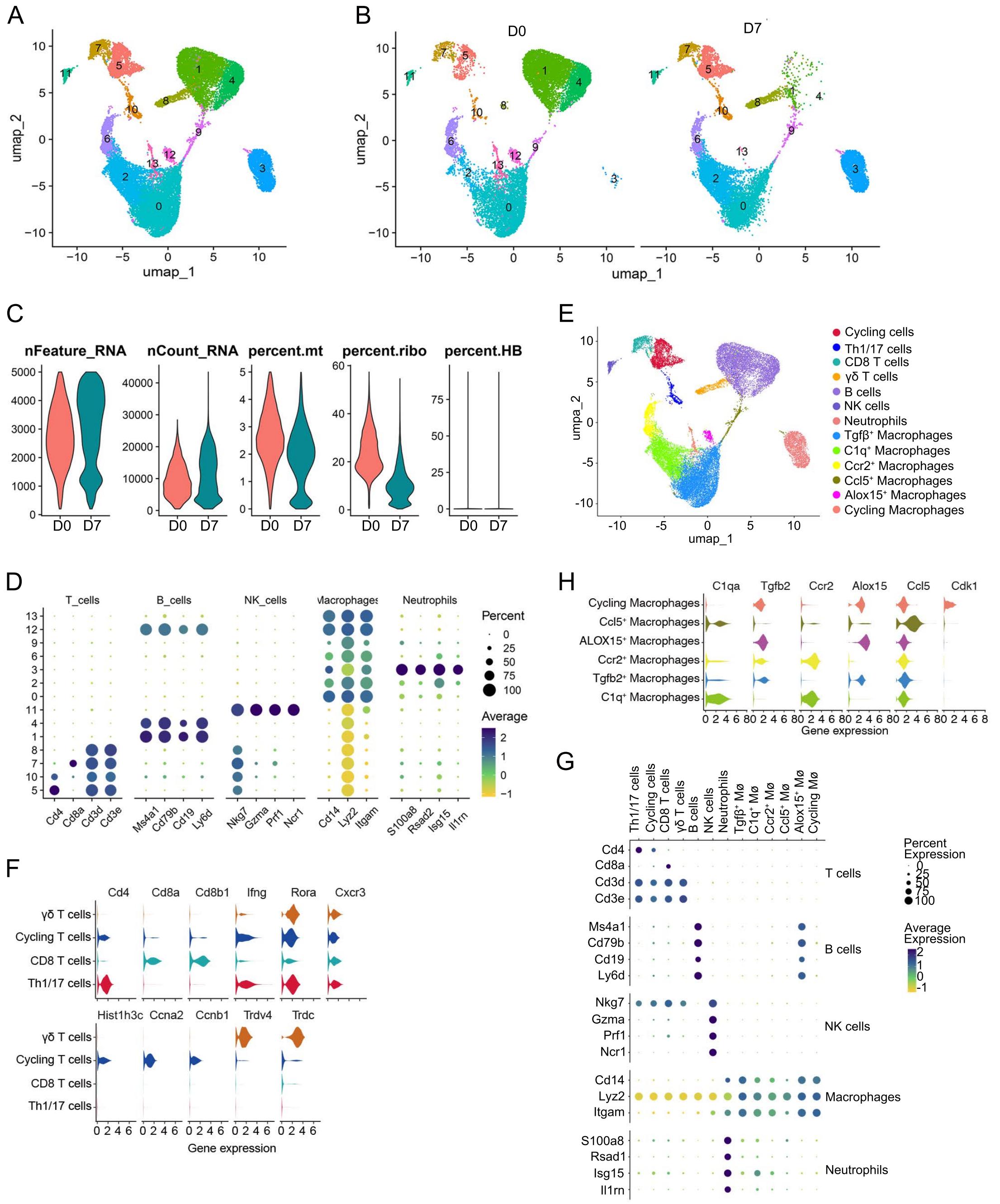

Figure S4. Quality control of the single cell RNA sequencing data from BCG-induced tuberculous pleurisy mouse models.

(A) Umap plot showed the Seurat clusters of single cell RNA sequencing data. (B) Umap plot of the Seurat clusters split by the samples. (C) Violin plot showed the numbers of genes and Unique Molecular Identifiers (UMIs), and the percentages of mitochondrial genes, ribosome genes and hemoglobin genes of single cell RNA sequencing data. (D) Dot plot showed the average expression of the known characteristic genes of immune cells within Seurat clusters. (E) Umap plot of cells isolated from pleural lavage fluid colored by the cell types. (F) The violin plot showed the average expression of the known characteristic genes of immune cells. (G) The violin plot indicated the expression of the marker genes in the corresponding T cell clusters. (H) The violin plot indicated the expression of the marker genes in the corresponding macrophage clusters.

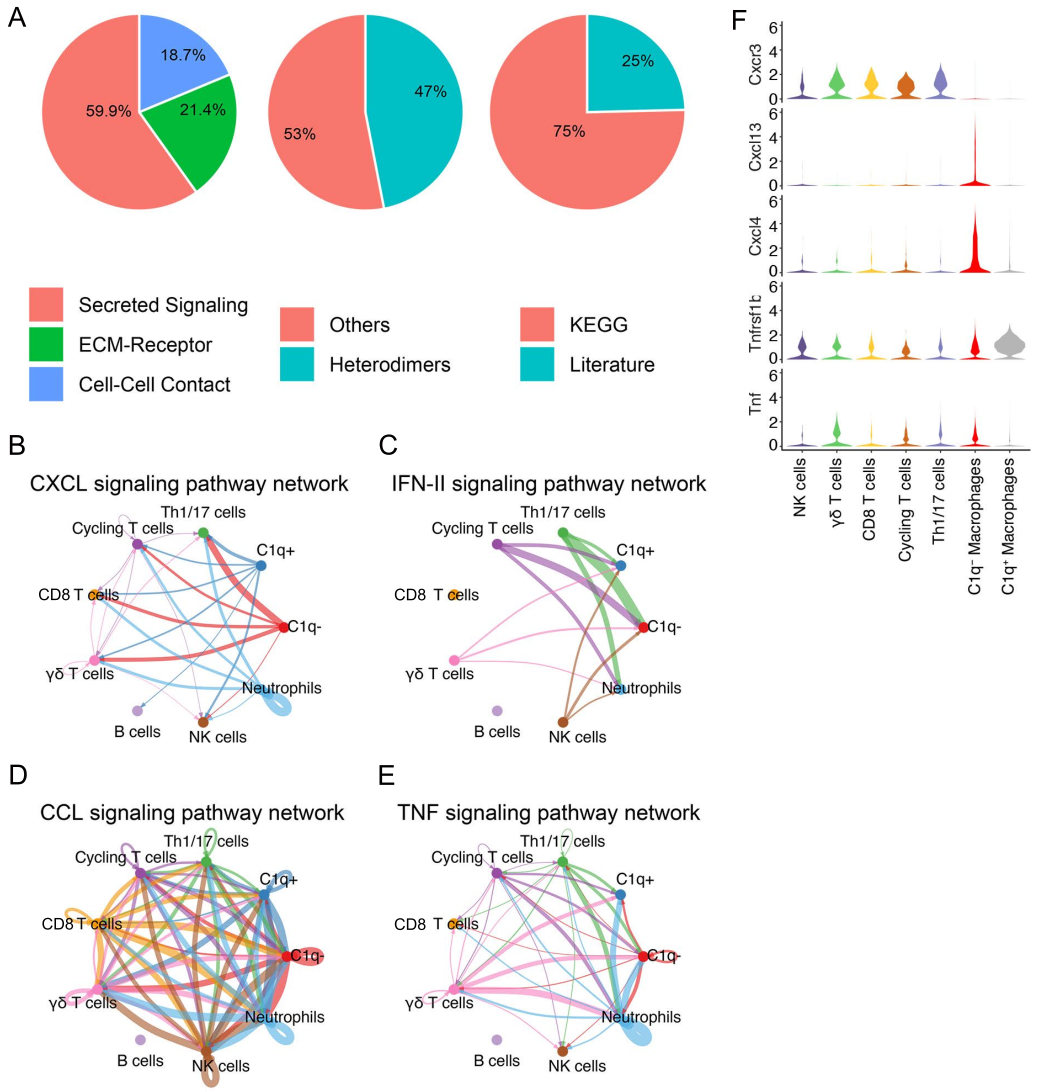

Figure S5. Cell-to-cell interaction of the single cell RNA sequencing data from BCG-induced tuberculous pleurisy mouse models.

(A) The pie chart showed the classification ratio of receptor-ligand pairs in the CellChat database. (B-E) The inferred networks of CXCL (B), IFN-II (C), CCL (D) and TNF (E) signaling pathways. Circle sizes were proportional to the number of cells in each cell group and edge width represented the communication probability. (F) The violin plot indicated the expression of the ligand genes and receptor genes of Tnf-Tnfrsf1b and Cxcl4/Cxcl13-Cxcr3 pairs in the corresponding cell cluster.

Table S1

	D0 (n=5)	D1 (n=5)	D7 (n=5)	D14 (n=5)
Total cells ($\times 10^6$/cavity)	1.3 ± 0.1	17.9 ± 1	10.3 ± 0.7	11.9 ± 0.8
Cell counts (%)				
Macrophages	41.9 ± 7.0	53.7 ± 3.0	34.0 ± 1.6	35.8 ± 1.1
Lymphocytes	34.6 ± 5.9	17.0 ± 3.1	62.4 ± 7.5	61.2 ± 6.4
